# Food anaphylaxis in the United Kingdom: analysis of national data, 1998-2018

**DOI:** 10.1136/bmj.n251

**Published:** 2021-02-17

**Authors:** Alessia Baseggio Conrado, Despo Ierodiakonou, M Hazel Gowland, Robert J Boyle, Paul J Turner

**Affiliations:** 1 National Heart and Lung Institute, Imperial College London, Norfolk Place, London W2 1PG, UK; 2 Health Planning Unit, Department of Social Medicine, Faculty of Medicine, University of Crete, Heraklion, Crete, Greece; 3 Allergy Action, St Albans, UK

## Abstract

**Objective:**

To describe time trends for hospital admissions due to food anaphylaxis in the United Kingdom over the past 20 years.

**Design:**

Analysis of national data, 1998-2018.

**Setting:**

Data relating to hospital admissions for anaphylaxis and deaths, and prescription data for adrenaline autoinjector devices.

**Participants:**

UK population as a whole and devolved nations (England, Scotland, Wales, and Northern Ireland).

**Main outcome measures:**

Time trends, age, and sex distributions for hospital admissions for anaphylaxis due to food and non-food triggers, and how these admission rates compare with the case fatality rate (number of fatalities as a proportion of hospital admissions).

**Results:**

Between 1998 and 2018, 101 891 people were admitted to hospital for anaphylaxis. Of these admissions, 30 700 (30.1%) were coded as due to a food trigger. Food anaphylaxis admissions increased from 1.23 to 4.04 per 100 000 population per year (from 1998 to 2018), an annual increase of 5.7% (95% confidence interval 5.5% to 5.9%, P<0.001). The largest increase in hospital admissions was observed in children younger than 15 years, with an increase from 2.1 to 9.2 admissions per 100 000 population per year (an annual increase of 6.6%, 95% confidence interval 6.3% to 7.0%). For comparison, the annual increase was 5.9% (5.6% to 6.2%) in people aged 15-59 years and 2.1% (1.8% to 3.1%) in those aged 60 years and older. 152 deaths were identified where the fatal event was probably caused by food induced anaphylaxis. The case fatality rate decreased from 0.7% to 0.19% for confirmed fatal food anaphylaxis (rate ratio 0.931, 95% confidence interval 0.904 to 0.959, P<0.001) and to 0.30% for suspected fatal food anaphylaxis (0.970, 0.945 to 0.996, P=0.024). At least 46% (86 of 187, which also includes 35 deaths in 1992-98) of deaths were triggered by peanut or tree nut. Cow’s milk was responsible for 17 of 66 (26%) deaths in school aged children. Over the same time period, prescriptions for adrenaline autoinjectors increased by 336% (estimated rate ratio 1.113, 95% confidence interval 1.112 to 1.113; an increase of 11% per year).

**Conclusions:**

Hospital admissions for food induced anaphylaxis have increased from 1998 to 2018, however the case fatality rate has decreased. In school aged children, cow’s milk is now the most common single cause of fatal anaphylaxis.

**Figure:**
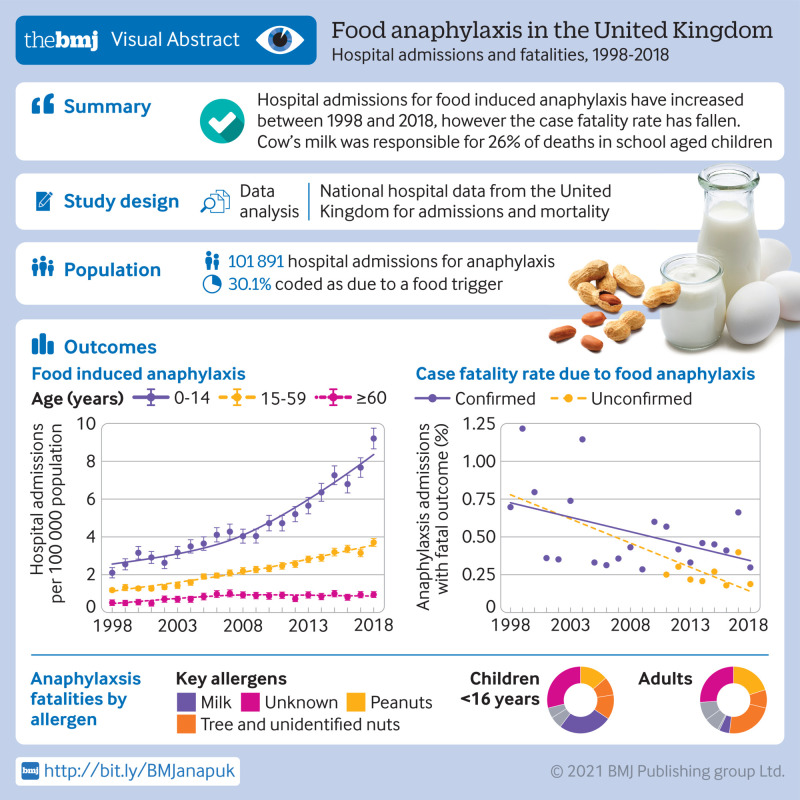


## Introduction

Food allergy continues to attract attention at a public health level and in the media, and is the commonest cause of potentially life threatening allergic reactions (anaphylaxis). Anaphylaxis is a serious systemic hypersensitivity reaction that is usually rapid in onset and could cause death.^1^ Substantial increases in hospital admissions due to food anaphylaxis have been reported globally^2^; however, whether this trend is continuing remains unclear, as does its association with the incidence of fatal reactions.

Mainstream media increasingly tend to report routinely collected data relating to food anaphylaxis,^3^ but often with little attempt to understand the issues and limitations of using such data. For example, admissions due to “allergy” might include day case admissions for allergy testing, irrespective of whether this results in an allergic reaction. As a consequence, these reports often contrast with the published data available in the scientific literature.

We have previously reported trends in anaphylaxis in England and Wales between 1992 and 2012.^4^ In this analysis, we extend these data up to and including 2018, and also report data from Northern Ireland and Scotland to provide a more comprehensive analysis of trends in hospital admissions in the United Kingdom, which can be matched to UK fatality data. We explore time trends in all cause and specific cause anaphylaxis events in different age groups and by sex, and evaluate changes in the case fatality rate (number of fatalities as a proportion of hospital admissions) for food induced anaphylaxis in the UK.

## Methods

Hospital admissions data for England and the devolved administrations (Scotland, Wales, and Northern Ireland) are collected in the Hospital Episodes Statistics database (coordinated through NHS Digital) for England, Patient Episode Database for Wales (NHS Wales Informatics Service), Information Services Division (NHS Scotland), and Hospital Activity Statistics (Health-NI), respectively. We extracted data from these datasets relating to hospital admissions from 1998 to 2018, where the primary diagnosis was anaphylaxis. Attendances to the emergency department that did not result in a hospital admission were excluded. ICD-10 (international classification of diseases, 10th revision) codes were used: anaphylactic shock due to adverse food reactions (T78.0); anaphylactic shock, unspecified (T78.2); and anaphylactic shock due to adverse effects of correct drug or medicament properly administered (T88.6). Admissions to hospital where a primary T78 code was associated with a secondary X23 code were classified as being caused by insect sting related anaphylaxis. We also included the following codes, which are used to code non-anaphylaxis admissions due to an allergic cause: other adverse food reactions, not elsewhere classified (T78.1); and other and unspecified allergy (T78.4). Because of data access restrictions, children consisted of young people up to 15 years old.

### Fatal food induced anaphylaxis data

Deaths in England and Wales are recorded by a medical doctor, and these data are documented by the Office for National Statistics. Since 1992, anaphylaxis fatality data have also been collected by the UK Fatal Anaphylaxis Registry. We updated previously published data from the UK Fatal Anaphylaxis Registry^4^ by using inquest reports and reports in the national media; we used identical methods because the attribution and coding of deaths can be unreliable. For each death, the probability that it was caused by food induced anaphylaxis was assessed; deaths caused by an acute asthma exacerbation were included only when strong evidence existed that the episode was triggered by an identified allergen to which the deceased patient had a known allergy. Data were cross checked with the Office for National Statistics database when sufficient information was available to determine the likely cause of death. While we included reports of fatal food anaphylaxis for people living in Scotland and Northern Ireland, we were unable to cross check these data with the equivalent databases in these nations.

### Adrenaline autoinjector prescriptions

We obtained annual prescriptions data for adrenaline autoinjectors from NHS Digital (for NHS England), Public Health Scotland, NHS Wales Shared Services Partnership, and Health and Social Care Northern Ireland Business Services Organisation.

### Statistical analyses

Hospital admissions data were expressed per 100 000 population for the equivalent Office for National Statistics population estimate for that year. We calculated age specific rates of hospital admissions and fatalities by standardising to the age distribution of the population to mid-2009 estimates as reported by the Office for National Statistics. We used Poisson regression to estimate the rate ratio (multiplicative increase of the rate per year over the study period) and 95% confidence intervals for the annual increase in rates, as previously described.^4^ We estimated rate ratios for the overall study period (1998-2018), but also the most recent five and 10 year periods to determine possible changes in time trends. A rate ratio of 1.0 implies no annual change in rate, and a 95% confidence interval that includes 1.0 indicates the observed rate ratio is not statistically significant. All statistical analyses were run in the programming language R.

### Patient and public involvement

No patients were involved in setting the research question or the outcome measures, however input was obtained from representatives from Allergy UK and the Anaphylaxis Campaign to inform the presentation of the data included in this paper.

## Results

Hospital admissions due to allergy have increased steadily across all ages between 1998 and 2018, from 10.0 to 28.0 admissions per 100 000 population per year, an increase of 179% (supplementary fig 1), with an estimated rate ratio of 1.043 (95% confidence interval 1.042 to 1.043, P<0.001). Between 1998 and 2018, there were 255 913 admissions, of which 101 891 (39.8%) were coded as (all cause) anaphylaxis; the remainder also included day cases (for example, allergy testing such as food challenges). When we limited the analysis to anaphylaxis (codes T78.0, T78.2, T88.6), hospital admissions increased by 179%, from 4.1 to 11.5 per 100 000 population per year, equivalent to a rate ratio of 1.047 (95% confidence interval 1.046 to 1.048, P<0.001). No obvious impact was noted after maximum waiting time targets in emergency departments were introduced by the UK government in 2004; this finding is consistent with our previous data.^4^

**Fig 1:**
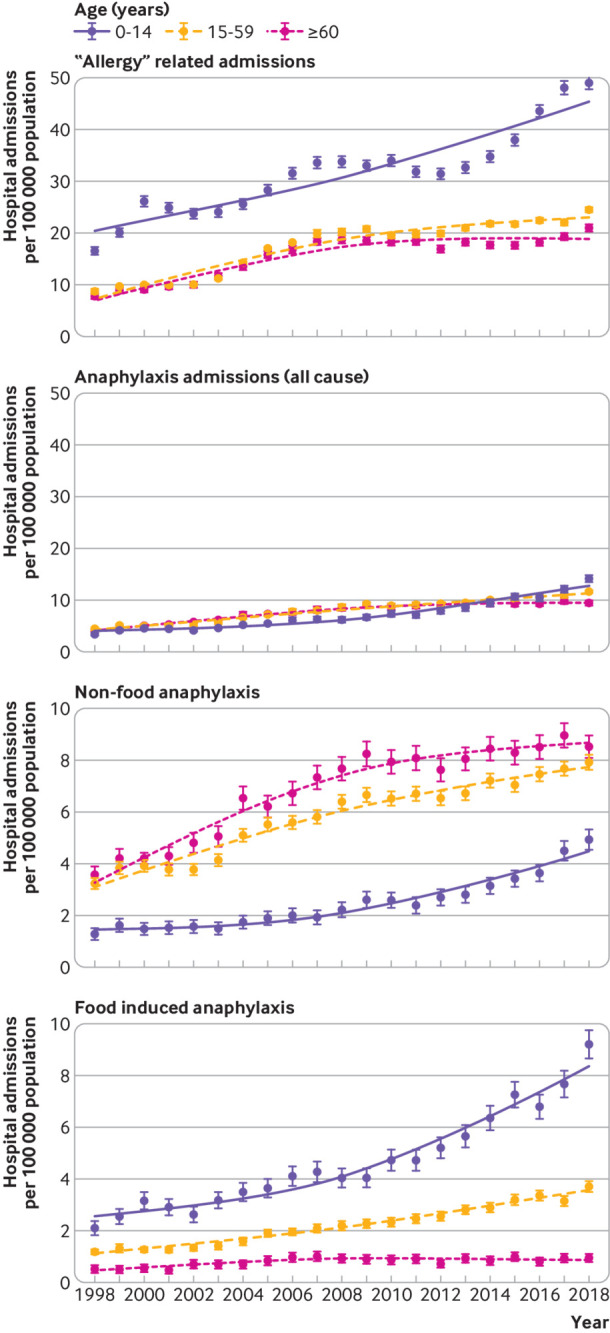
Time trends in hospital admissions per 100 000 population from 1998 to 2018 by age. From upper panel downwards: admissions due to “allergy”; all cause anaphylaxis; non-food anaphylaxis; and food induced anaphylaxis. Vertical bars represent standard error of the mean

When we analysed these data by age group (0-14, 15-59, ≥60 years), a similar trend was observed, although we found evidence of a plateau in adult allergy admissions since 2014, but not for anaphylaxis (fig 1, upper two panels). Figure 1 (third panel) shows hospital admissions for anaphylaxis due to a non-food trigger or when the trigger was unknown. Across all age groups, age specific rates for hospital admissions increased: rate ratios 1.065 (95% confidence interval 1.060 to 1.069) for 0-14 years, 1.041 (1.039 to 1.042) for 15-59 years, and 1.050 (1.048 to 1.053) for age 60 years and older.

### Hospital admissions due to food induced anaphylaxis

Between 1998 and 2018, there were 101 891 admissions for anaphylaxis, of which 30 700 (30.1%) were coded as due to a food trigger. These admissions increased over the study period from 1.23 to 4.04 admissions per 100 000 population per year (rate ratio 1.057, 95% confidence interval 1.055 to 1.059, P<0.001; supplementary fig 1). The greatest increase in admissions due to food anaphylaxis was observed for people younger than 15 years: from 2.1 to 9.2 admissions per 100 000 population per year (an increase of 339%), equivalent to a year-on-year increase of 6.6% (rate ratio 1.066, 1.063 to 1.070, P<0.001). The equivalent increase was 214% (1.059, 1.056 to 1.062, P<0.001) for people aged 15-59 years, and 78% (1.021, 1.02 to 1.03, P<0.001) for those aged 60 years or older over the same time period (fig 1, lower panel).

In 2011, the National Institute for Health and Care Excellence published guidance that recommended children younger than 16 years should be admitted to hospital under the care of a paediatric medical team after emergency treatment for suspected anaphylaxis.^5^ Therefore, we calculated rate ratios for the increase in hospital admissions due to food anaphylaxis in children for the time periods 1998-2010 (before guidance), 2011-18, and 2014-18. The rate ratios for these time periods were 1.057 (95% confidence interval 1.049 to 1.065), 1.091 (1.079 to 1.103), and 1.086 (1.064 to 1.109), respectively. These data imply that there was a substantial increase in hospital admissions after 2011, but also that the rate of increase has persisted from 2014 onwards. Therefore, the increase over the past five years cannot be attributed to the impact of the guidance.

### Association between sex and anaphylaxis hospital admission rates

**Fig 2:**
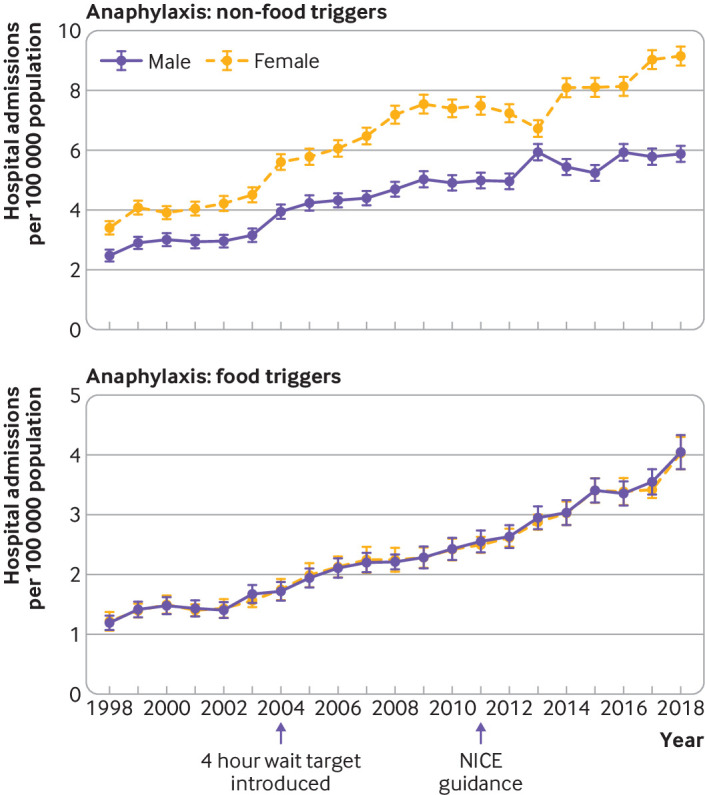
Impact of sex on hospital admissions for anaphylaxis due to non-food and food triggers. A national four hour target for accident and emergency visits was introduced in 2004. NICE (National Institute for Health and Care Excellence) guidance recommending overnight admission for paediatric anaphylaxis was introduced in 2011. Vertical bars represent standard error of the mean

**Fig 3:**
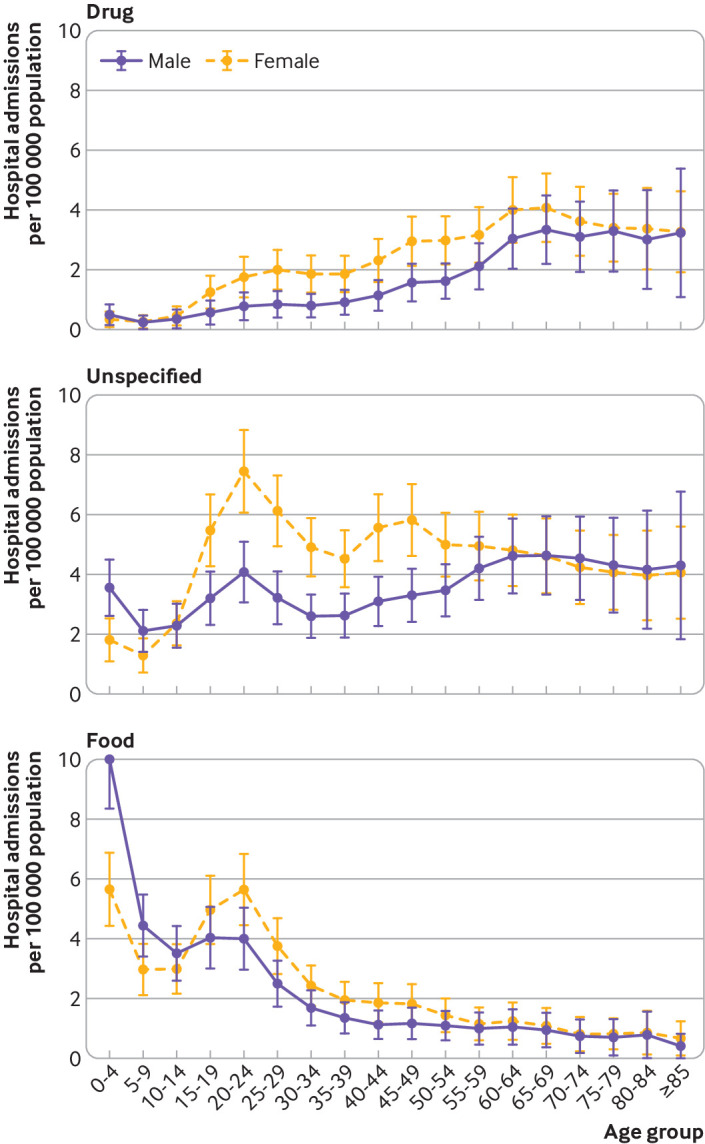
Sex differences by age for hospital admissions for anaphylaxis due to drug, unspecified, and food triggers. Vertical bars represent standard error of the mean

We observed a clear female predominance for non-food triggers of anaphylaxis admissions throughout the study period (fig 2, upper panel), whereas this trend was not apparent for food anaphylaxis (fig 2, lower panel). On further analysis, when we looked at sex differences by age, a clear male predominance was found in food anaphylaxis admissions before puberty (male-to-female ratio 1.6:1), which then reversed from age 15 years onwards (fig 3, lower panel). These findings are consistent with previous data.^6^ In contrast, there was a clear female predominance for drug induced anaphylaxis (fig 3, upper panel). The sex ratio for anaphylaxis admissions due to an unspecified trigger in younger children (fig 3, middle panel) implies that many of these admissions might have been due to a food trigger but were coded incorrectly.

### Time trends for anaphylaxis admissions in the UK by devolved nation

We analysed hospital admissions by devolved nation and observed a lower rate of admissions per 100 000 in Wales compared with the other devolved nations for all cause anaphylaxis (supplementary fig 2). Because of data protection issues, we were unable to assess the impact of Welsh residents seeking medical attention in English hospitals, which might explain this finding. For food induced anaphylaxis, hospital admissions were higher in England and Scotland than in Northern Ireland and Wales, although these differences were not significant. The annual increase in admissions showed evidence of plateauing in Wales and Northern Ireland, as reflected by the estimated rate ratio for the year-on-year increase in admissions when considering the period 2014-18 (table 1). However, the confidence intervals for these data are wider because of the lower number of admissions for anaphylaxis in Wales (3.7%) and Northern Ireland (3.2%).

**Table 1:** Time trends in hospital admissions for all cause and food induced anaphylaxis during 1998-2018, 2009-18, and 2014-18 by devolved nation. Data are estimated rate ratios (95% confidence intervals)

Time period and nation	All cause anaphylaxis	Food induced anaphylaxis
**1998-2018**		
UK	1.047 (1.046 to 1.048)	1.057 (1.055 to 1.059)
England	1.052 (1.051 to 1.053)	1.061 (1.058 to 1.063)
Scotland	1.030 (1.027 to 1.034)	1.046 (1.040 to 1.053)
Wales	1.009 (1.004 to 1.015)	1.015 (1.005 to 1.024)
Northern Ireland	1.018 (1.012 to 1.024	1.039 (1.027 to 1.052)
**2009-18**		
UK	1.036 (1.033 to 1.039)	1.063 (1.058 to 1.068)
England	1.039 (1.036 to 1.042)	1.063 (1.057 to 1.069)
Scotland	1.011 (1.002 to 1.021)	1.072 (1.054 to 1.090)
Wales	1.014 (0.998 to 1.030)	1.049 (1.020 to 1.078)
Northern Ireland	1.033 (1.017 to 1.050)	1.045 (1.011 to 1.080)
**2014-18**		
UK	1.042 (1.034 to 1.050)	1.062 (1.048 to 1.076)
England	1.047 (1.039 to 1.056)	1.069 (1.054 to 1.085)
Scotland	1.034 (1.007 to 1.062)	1.038 (0.992 to 1.085)
Wales	0.965 (0.924 to 1.008)	0.981 (0.911 to 1.056)
Northern Ireland	0.988 (0.945 to 1.032)	0.991 (0.907 to 1.082)

### Fatal food induced anaphylaxis

**Fig 4:**
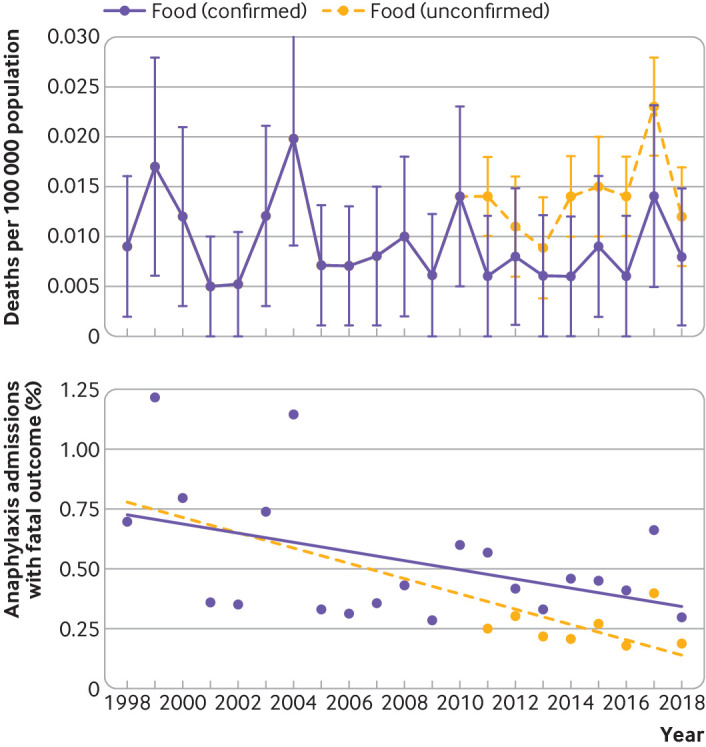
Upper panel: deaths due to food induced anaphylaxis per 100 000 population, 1998-2018. Lower panel: case fatality rate for food induced anaphylaxis (with line fitted using linear regression), 1998-2018. Vertical bars represent standard error of the mean

**Fig 5:**
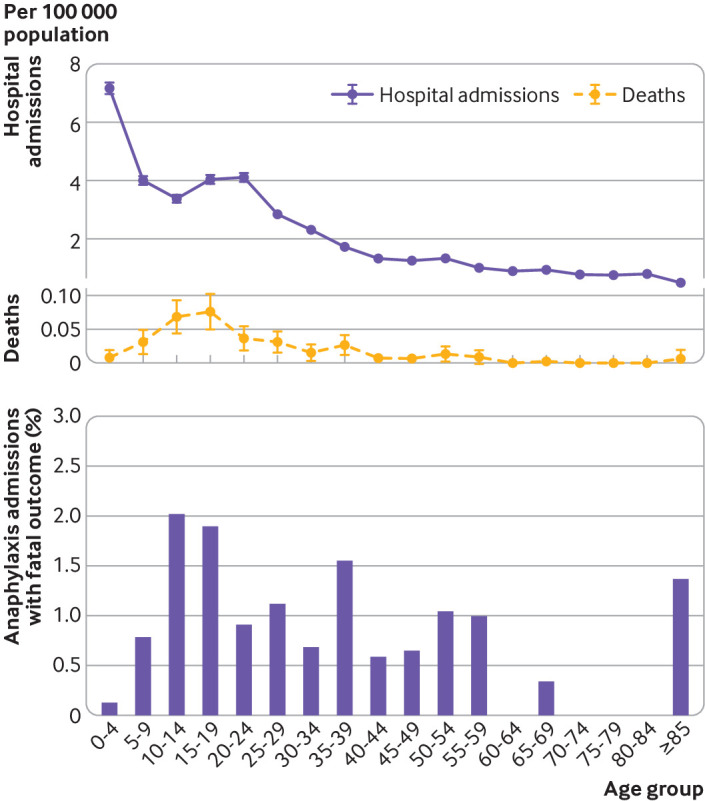
Upper panel: hospital admissions and deaths due to food induced anaphylaxis by age group per 100 000 population, 1998-2018. Lower panel: case fatality rate for food induced anaphylaxis by age group, 1998-2018. Vertical bars represent standard error of the mean

Between 1998 and 2018, we identified 152 deaths where there was a high level of suspicion that death was due to food induced anaphylaxis. For 120 deaths, the coronial inquiry found the cause was food induced anaphylaxis; for the remaining 32 deaths, sufficient information from the inquest or other sources was reported that death had been associated with exposure to a food allergen to which the person was allergic. The annual fatality rate due to food induced anaphylaxis was 0.009 per 100 000 population in 1998 and 0.008 per 100 000 population in 2018 (fig 4, upper panel). The case fatality rate decreased from 0.70% in 1998 to 0.19% (confirmed fatal food anaphylaxis) or 0.30% (suspected fatal food anaphylaxis) in 2018 (fig 4, lower panel). The estimated rate ratio was 0.931 (95% confidence interval 0.904 to 0.959, P<0.001) for confirmed fatal food anaphylaxis and 0.970 (0.945 to 0.996, P=0.024) for suspected fatal food anaphylaxis. The age distribution for fatal food anaphylaxis peaked during teenage years (fig 5, upper panel); this age group also showed a peak in hospital admissions. When we assessed the case fatality rate by age group, the highest fatality rate was observed during teenage years, but remained raised throughout adulthood (fig 5, lower panel). Although children younger than five years old were most likely to be admitted to hospital with anaphylaxis, the rate of deaths in this age group was low.

**Fig 6:**
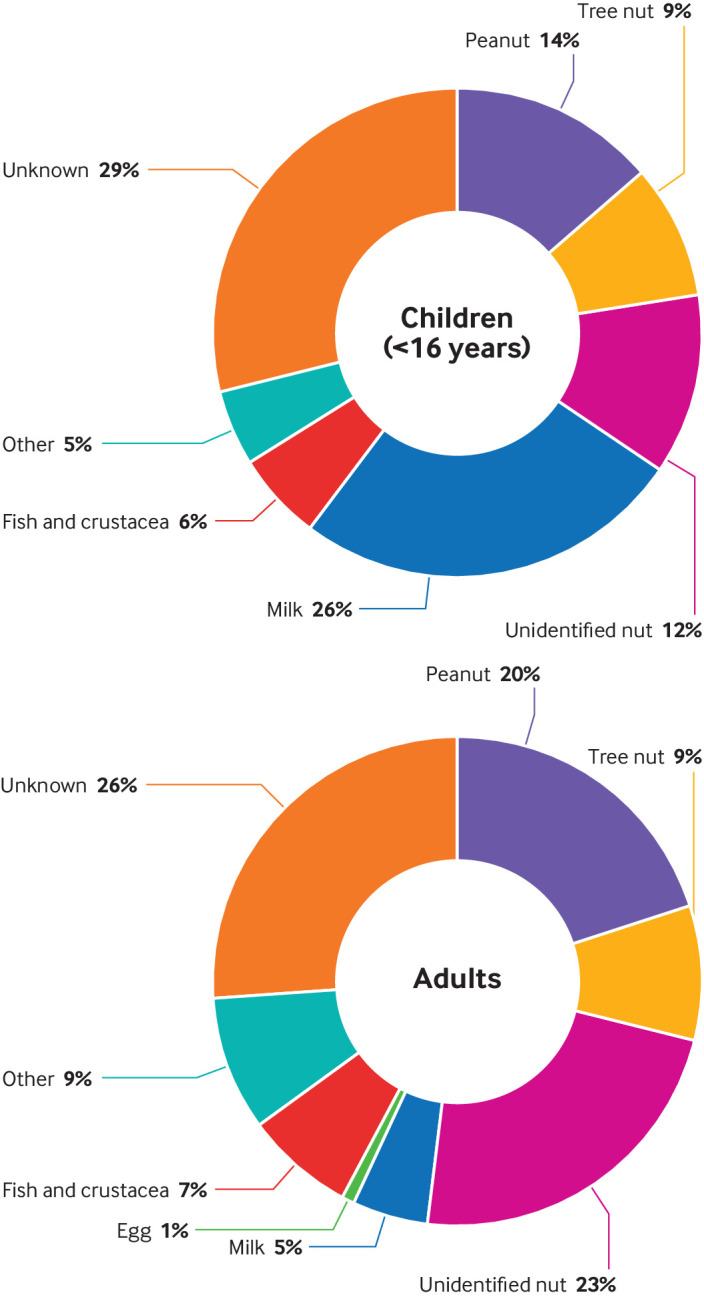
Cause of fatal food induced anaphylaxis by trigger in children (younger than 16) and adults, 1992-2018

**Fig 7:**
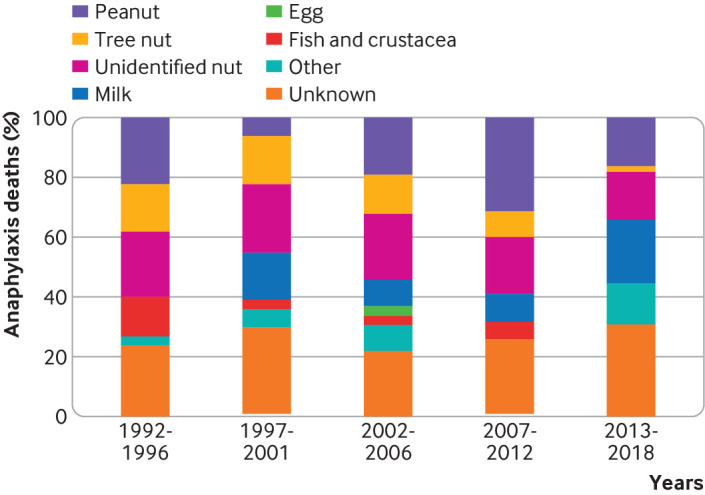
Changes in the relative proportions of fatal food induced anaphylaxis by specific foods, 1992-2018

Figure 6 and figure 7 show the triggers for fatal food induced anaphylaxis, and include a further 35 deaths that occurred between 1992 and 1998.^4^ In more than a quarter of these events, the precise food could not be identified. At least 86 (46%) deaths were triggered by peanut or tree nut. However, cow’s milk was responsible for 26% of deaths in children and 5% in adults, despite allergy to cow’s milk being uncommon in older children and adults. Since 1992, a downward trend has occurred in the proportion of deaths due to peanut or tree nut, but deaths due to cow’s milk exposure have increased (fig 7).

### Prescription of adrenaline autoinjector devices

**Fig 8:**
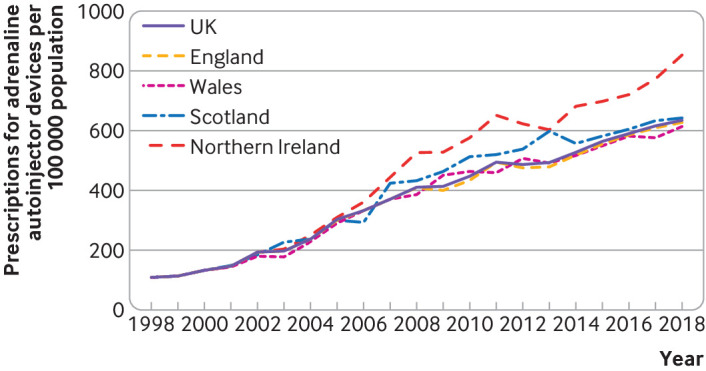
Prescription of adrenaline autoinjectors to UK residents from 1998 to 2018, overall and by devolved nation. Data refer to number of prescriptions issued (rather than number of devices)

Figure 8 shows time trends in the prescription of adrenaline autoinjector devices to UK residents, overall and by devolved nation. Overall, prescriptions increased by 336% from 1998 to 2018, with an estimated rate ratio of 1.113 (95% confidence interval 1.112 to 1.113) over the study period. This figure represents a year-on-year increase of 11% per year.

## Discussion

In this analysis of hospital admissions and deaths due to food anaphylaxis over a 20 year period, we have shown that while hospital admissions have continued to increase across all age groups, deaths have not. Furthermore, over the same time period, the case fatality rate has more than halved, from 0.7% in 1998 to 0.3% in 2018. These data extend previous findings reported for England and Wales to the whole of the UK population,^4^ and are consistent with epidemiological trends in hospital admissions for anaphylaxis in the United States and Australia.^2^

Improvements in the recognition and management of anaphylaxis could partly explain the decrease in the case fatality rate despite increasing hospital admissions for anaphylaxis. No evidence exists to suggest that the clinical criteria used to diagnose anaphylaxis have changed in the UK over the study period. The introduction of national guidance from the National Institute for Health and Care Excellence in 2011 probably resulted in a small increase in hospital admissions due to anaphylaxis, but the year-on-year increase has persisted since then. Whether a true increase in the prevalence of anaphylaxis has occurred (rather than a reduction in the threshold to admit patients presenting with anaphylaxis) is unclear because evidence is lacking for an increase in prevalence of food allergy in the UK (and elsewhere) over the same time period.^7^

Our data represent a large published series of food anaphylaxis fatalities in the literature, and clearly show that while preschool aged children have the highest rate of hospital admissions for anaphylaxis, the case fatality rate in this age group is low. Teenagers are considered to be at highest risk of fatal reactions,^4^ ^8^ but our data show that while this is true, the case fatality rate remains increased well into mid-adulthood. These findings challenge the traditional view that teenagers are more at risk of fatal outcomes because of risk taking behaviours,^8^ and support our hypothesis that there might be a specific, age dependent vulnerability to severe outcomes from food induced allergic reactions in early to mid-adulthood.^4^

We found that over the past 25 years, the proportion of fatalities due to peanut or tree nut has fallen (which we attribute to increased awareness of nut allergies by food businesses). In contrast, we observed a concerning increase in fatalities due to cow’s milk, which is now the most common cause of fatal food anaphylaxis in children in the UK. This pattern has also been noted in North America and Israel.^9^ ^10^ ^11^ Although cow’s milk allergy is relatively uncommon in adults, it was still responsible for 5% of fatal food reactions in adults in the UK. While there is increasing recognition of the risks posed by peanut and tree nut allergies, this is not true for cow’s milk allergy.^12^ ^13^ Dairy foods form an important part of Western diets. Cow’s milk has a relatively high protein content, so very low levels of exposure can be sufficient to cause reactions. Allergen control is further complicated by the possibility of homogenous distribution (where the allergen is equally dispersed throughout the food product), as opposed to particulate contamination, which is typical for nuts.^14^ In general, cow’s milk protein allergy in young children is less severe, and most infants with cow’s milk protein allergy outgrow their allergy in early childhood.^15^ However, in older children with persisting milk allergy, it is clearly a common cause of anaphylaxis and life threatening reactions. These people tend to have other concomitant atopic diseases including asthma, which may increase the risk of severe reactions.^12^ ^15^

### Strengths and limitations

We use a national dataset in the context of the UK health system, which provides a unique opportunity to draw robust conclusions on changes in hospital admissions and outcomes due to food anaphylaxis. In other countries, data collection might be less systematic and not applied to the entire population. However, our data do have some limitations, as previously discussed elsewhere,^2^ ^4^ ^16^ including miscoding (for the trigger and for misdiagnosis, with non-anaphylaxis reactions being miscoded as anaphylaxis). By using the same methods throughout the study period, we were able to monitor time trends irrespective of the risk of miscoding, which is unlikely to have been affected by time. Unfortunately, we could not include patients with anaphylaxis treated in emergency departments who did not require hospital admission because these datasets are incomplete and prone to miscoding.^4^

Our data highlight some of the potential pitfalls in analysing health datasets in terms of anaphylaxis admissions. Hospital admissions for anaphylaxis are often used as a surrogate measure for the occurrence of anaphylaxis, but this is a false assumption. In a UK analysis, González-Pérez and colleagues reported that less than half of anaphylaxis episodes present to emergency departments.^17^ There is little evidence that the incidence of food allergy or anaphylaxis has increased over the past decade in the UK^7^; our observation that prescriptions for adrenaline autoinjectors have increased at an annual rate of 11% (despite hospital admissions for anaphylaxis increasing at around half that rate over the same period) should not be interpreted as implying an increase in the prevalence of people at risk of anaphylaxis. In the UK, hospital admissions due to allergy also include elective diagnostic procedures such as oral food challenges; only around 12% of admissions for allergy are due to food anaphylaxis.

Due to the limitations of ICD-10 codes, food related anaphylaxis is often coded as anaphylactic shock, despite shock being a rare occurrence in food anaphylaxis.^2^ ^4^ ^16^ Therefore, no conclusions can be drawn as to the severity of anaphylaxis when analysing hospital admissions, unless other considerations are taken into account (such as admission to intensive care or fatal outcomes). The introduction of ICD-11 will hopefully address many of the current limitations of ICD-10,^18^ although a proposal to include a classification of anaphylaxis by clinical severity was rejected.^19^

### Conclusions and policy implications

The case fatality rate due to food anaphylaxis fell between 1998 and 2018 in the UK, despite a threefold increase in hospital admissions for food anaphylaxis over the same period. Cow’s milk is increasingly identified as the culprit allergen for fatal food reactions, and is now the commonest cause of fatal anaphylaxis in children. More education is needed to highlight the specific risks posed by cow’s milk to people who are allergic to increase awareness among food businesses.^15^ Finally, further work is needed to assess the evidence for an age related vulnerability to severe anaphylaxis in young adults, therefore improving our ability to risk stratify patients with food allergies and to reduce the risk of fatal outcomes.

**What is already known on this topic**

Hospital admissions for food anaphylaxis are increasing globally, and have doubled between 1998 and 2012 in England and Wales
Over the same time period, the incidence of fatal anaphylaxis has remained stable

**What this study adds**

Despite an increase in hospital admissions for food induced anaphylaxis in the UK between 1998 and 2018, the case fatality rate has decreased
Most young children with allergy to cow’s milk will outgrow their allergy; however, in those with persisting allergy, cow’s milk is responsible for more than a quarter of deaths caused by food anaphylaxis
Prescriptions for adrenaline autoinjectors have increased at almost double the rate of hospital admissions; the impact of this increase on fatal outcomes is unclear

## Web extra

Extra material supplied by authors

Web appendix: supplementary figures

## Notes

Ethical approval: Ethical approval was not required.

Data sharing: Requests for data can be made to the corresponding author.

The lead author affirms that the manuscript is an honest, accurate, and transparent account of the study being reported. No important aspects of the study have been omitted, and all discrepancies from the study as planned have been explained.

Dissemination to participants and related patient and public communities: Findings of this study have been disseminated through the British Society for Allergy and Clinical Immunology, and to the Anaphylaxis Working Group at the Resuscitation Council UK. Additionally, further dissemination is planned through presentations at medical conferences, press releases, social media, and on institutional websites upon publication.

Provenance and peer review: Not commissioned; externally peer reviewed.
