# Supplementary material for: Food anaphylaxis in the United Kingdom: analysis of national data, 1998-2018
Source: BMJ. 2021 Feb 17;372:n251. doi: 10.1136/bmj.n251 (PMC7885259; doi:10.1136/bmj.n251)
Supplement: Supplementary file 1 — Web appendix: supplementary figures [file basc061604.ww.pdf]

Food Anaphylaxis in the UK: an analysis of national data, 1998-2018

Alessia Baseggio Conrado, Despo Ierodiakonou, M. Hazel Gowland,

Robert J. Boyle, Paul J. Turner

SUPPLEMENTARY FIGURES

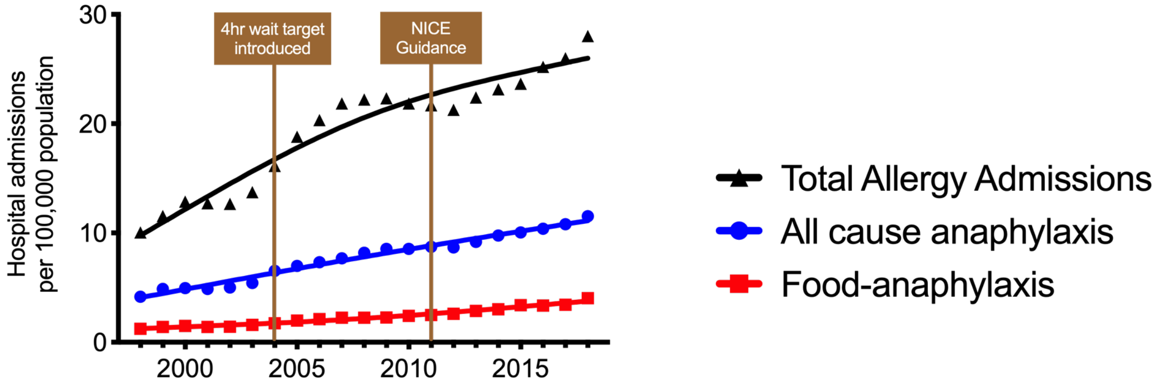

Suppl. Figure 1: Hospital admissions for "allergy", all-cause anaphylaxis and food-induced anaphylaxis in the United Kingdom from 1998-2018.

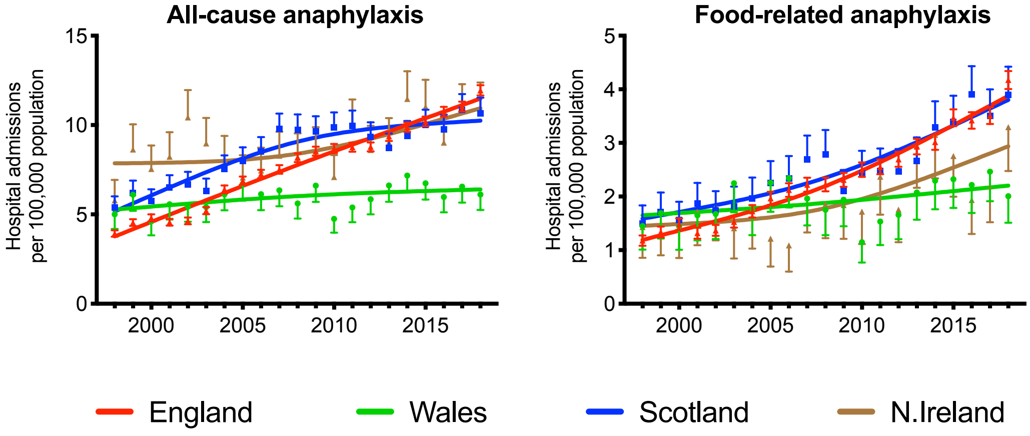

Suppl. Figure 2: Time trends in anaphylaxis admissions by devolved nation. Vertical bars represent SEMs.
